# Supplementary material for: Multiomics Investigation of Exhausted T Cells in Glioblastoma Tumor Microenvironment: CCL5 as a Prognostic and Therapeutic Target
Source: Int J Mol Sci. 2025 Oct 12;26(20):9920. doi: 10.3390/ijms26209920 (PMC12563952; doi:10.3390/ijms26209920)
Supplement: Supplementary file 1 [file ijms-26-09920-s001.zip › Supplementary.pdf]

Supplementary Materials

This PDF file includes:

Tables. S1 to S6

Figures. S1 to S4

Details of in vitro experiment

PART OF TABLES

TABLE S1. Complete results of Mendelian randomization

outcome	exposure	Method	nsnp	b	se	*P*
CD28- CD8+ T cell %CD8+ T cell	NHGRI-EBI GWAS catalog	Weighted median	27	-0.0347	0.0375	0.3539
CD28- CD8+ T cell %CD8+ T cell	NHGRI-EBI GWAS catalog	Inverse variance weighted	27	-0.0471	0.0255	0.0652
CD28- CD8+ T cell %CD8+ T cell	NHGRI-EBI GWAS catalog	Simple mode	27	0.0027	0.0595	0.9644
CD28- CD8+ T cell %CD8+ T cell	NHGRI-EBI GWAS catalog	Weighted mode	27	-0.0255	0.0383	0.5124
CD28- CD8+ T cell Absolute Count	NHGRI-EBI GWAS catalog	Weighted median	27	-0.0217	0.0396	0.5841
CD28- CD8+ T cell Absolute Count	NHGRI-EBI GWAS catalog	Inverse variance weighted	27	-0.0483	0.0299	0.1062
CD28- CD8+ T cell Absolute Count	NHGRI-EBI GWAS catalog	Simple mode	27	-0.0445	0.0737	0.5510
CD28- CD8+ T cell Absolute Count	NHGRI-EBI GWAS catalog	Weighted mode	27	-0.0116	0.0415	0.7815
CD45RA- CD28- CD8+ T cell Absolute Count	NHGRI-EBI GWAS catalog	Weighted median	27	-3.4910	4.4580	0.4335
CD45RA- CD28- CD8+ T cell Absolute Count	NHGRI-EBI GWAS catalog	Inverse variance weighted	27	-5.3750	3.2070	0.0937
CD45RA- CD28- CD8+ T cell Absolute Count	NHGRI-EBI GWAS catalog	Simple mode	27	-10.9100	7.2140	0.1426
CD45RA- CD28- CD8+ T cell Absolute Count	NHGRI-EBI GWAS catalog	Weighted mode	27	-6.4130	4.7930	0.1925
CD45RA- CD28- CD8+ T cell %CD8+ T cell	NHGRI-EBI GWAS catalog	Weighted median	27	-0.3824	0.4779	0.4237
CD45RA- CD28- CD8+ T cell %CD8+ T cell	NHGRI-EBI GWAS catalog	Inverse variance weighted	27	-0.7579	0.3340	0.0233
CD45RA- CD28- CD8+ T cell %CD8+ T cell	NHGRI-EBI GWAS catalog	Simple mode	27	-0.5595	0.7163	0.4418
CD45RA- CD28- CD8+ T cell %CD8+ T cell	NHGRI-EBI GWAS catalog	Weighted mode	27	-0.3754	0.4757	0.4372
CD28- CD8+ T cell %CD8+ T cell	UCSF/Mayo	Weighted median	3	-0.1150	0.0581	0.0477
CD28- CD8+ T cell %CD8+ T cell	UCSF/Mayo	Inverse variance weighted	3	-0.0964	0.0481	0.0449
CD28- CD8+ T cell %CD8+ T cell	UCSF/Mayo	Simple mode	3	-0.1166	0.0697	0.2361
CD28- CD8+ T cell %CD8+ T cell	UCSF/Mayo	Weighted mode	3	-0.1158	0.0680	0.2307
CD28- CD8+ T cell Absolute Count	UCSF/Mayo	Weighted median	3	-0.1066	0.0642	0.0966
CD28- CD8+ T cell Absolute Count	UCSF/Mayo	Inverse variance weighted	3	-0.0990	0.0535	0.0642
CD28- CD8+ T cell Absolute Count	UCSF/Mayo	Simple mode	3	-0.1118	0.0769	0.2835
CD28- CD8+ T cell Absolute Count	UCSF/Mayo	Weighted mode	3	-0.1093	0.0696	0.2572
CD45RA- CD28- CD8+ T cell Absolute Count	UCSF/Mayo	Weighted median	3	-11.3929	6.5557	0.0822
CD45RA- CD28- CD8+ T cell Absolute Count	UCSF/Mayo	Inverse variance weighted	3	-11.9088	5.6194	0.0341
CD45RA- CD28- CD8+ T cell Absolute Count	UCSF/Mayo	Simple mode	3	-12.8612	7.4958	0.2283
CD45RA- CD28- CD8+ T cell Absolute Count	UCSF/Mayo	Weighted mode	3	-9.833	7.4966	0.3200
CD45RA- CD28- CD8+ T cell %CD8+ T cell	UCSF/Mayo	Weighted median	3	-1.1311	0.7618	0.1376
CD45RA- CD28- CD8+ T cell %CD8+ T cell	UCSF/Mayo	Inverse variance weighted	3	-1.0279	0.6422	0.1095
CD45RA- CD28- CD8+ T cell %CD8+ T cell	UCSF/Mayo	Simple mode	3	-1.3625	0.9353	0.2825
CD45RA- CD28- CD8+ T cell %CD8+ T cell	UCSF/Mayo	Weighted mode	3	-1.2047	0.8935	0.3100

CD28- CD8+ T cell %CD8+ T cell	GICC_GBM	Weighted median	12	-0.0424	0.0336	0.2075
CD28- CD8+ T cell %CD8+ T cell	GICC_GBM	Inverse variance weighted	12	-0.0547	0.0256	0.0329
CD28- CD8+ T cell %CD8+ T cell	GICC_GBM	Simple mode	12	-0.1297	0.0511	0.0277
CD28- CD8+ T cell %CD8+ T cell	GICC_GBM	Weighted mode	12	-0.0285	0.0462	0.5497
CD28- CD8+ T cell Absolute Count	GICC_GBM	Weighted median	12	-0.0606	0.036	0.0923
CD28- CD8+ T cell Absolute Count	GICC_GBM	Inverse variance weighted	12	-0.0603	0.0348	0.0833
CD28- CD8+ T cell Absolute Count	GICC_GBM	Simple mode	12	-0.109	0.0549	0.0724
CD28- CD8+ T cell Absolute Count	GICC_GBM	Weighted mode	12	-0.0748	0.0466	0.1369
CD45RA- CD28- CD8+ T cell Absolute Count	GICC_GBM	Weighted median	12	-3.6218	3.8646	0.3487
CD45RA- CD28- CD8+ T cell Absolute Count	GICC_GBM	Inverse variance weighted	12	-4.2093	3.108	0.1756
CD45RA- CD28- CD8+ T cell Absolute Count	GICC_GBM	Simple mode	12	-6.6669	6.2939	0.3122
CD45RA- CD28- CD8+ T cell Absolute Count	GICC_GBM	Weighted mode	12	-4.7288	5.0831	0.3722
CD45RA- CD28- CD8+ T cell %CD8+ T cell	GICC_GBM	Weighted median	12	-0.1877	0.4341	0.6655
CD45RA- CD28- CD8+ T cell %CD8+ T cell	GICC_GBM	Inverse variance weighted	12	-0.3285	0.3367	0.3293
CD45RA- CD28- CD8+ T cell %CD8+ T cell	GICC_GBM	Simple mode	12	-0.1016	0.6986	0.8869
CD45RA- CD28- CD8+ T cell %CD8+ T cell	GICC_GBM	Weighted mode	12	-0.0852	0.6464	0.8975

TABLE S2. Horizontal pleiotropy test of MR-Egger method.

outcome	exposure	egger_intercept	se	egger_*P*
CD45RA- CD28- CD8+ T cell %CD8+ T cell	NHGRI-EBI GWAS catalog	-0.263490815	0.15445223	0.100409082
CD28- CD8+ T cell %CD8+ T cell	UCSF/Mayo	-0.083841186	0.100321346	0.556817879
CD45RA- CD28- CD8+ T cell Absolute Count	UCSF/Mayo	1.790100224	11.71343901	0.903455896
CD28- CD8+ T cell %CD8+ T cell	GICC_GBM	-0.04382419	0.037534337	0.270062724

TABLE S3. The Cochran Q test on MR Egger method and IVW method.

outcome	exposure	Method	Q	Q_df	Q_*P*
CD45RA- CD28- CD8+ T cell %CD8+ T cell	NHGRI-EBI GWAS catalog	MR Egger	26.17341752	25	0.398390974
CD45RA- CD28- CD8+ T cell %CD8+ T cell	NHGRI-EBI GWAS catalog	Inverse variance weighted	29.22035131	26	0.301157287
CD28- CD8+ T cell %CD8+ T cell	UCSF/Mayo	MR Egger	0.013811915	1	0.906444746
CD28- CD8+ T cell %CD8+ T cell	UCSF/Mayo	Inverse variance weighted	0.712250336	2	0.700384949
CD45RA- CD28- CD8+ T cell Absolute Count	UCSF/Mayo	MR Egger	0.35806207	1	0.549584487
CD45RA- CD28- CD8+ T cell Absolute Count	UCSF/Mayo	Inverse variance weighted	0.381417392	2	0.826373279
CD28- CD8+ T cell %CD8+ T cell	GICC_GBM	MR Egger	11.24452575	10	0.338787319
CD28- CD8+ T cell %CD8+ T cell	GICC_GBM	Inverse variance weighted	12.77741738	11	0.308121958

TABLE S4. MCODE results for Cluster 1 and Cluster 2

MCODE.Clusters	MCODE.Node Status	MCODE.Score	Gene name
Cluster 1	Clustered	24.52272727	TRIP13
Cluster 1	Clustered	22.76129032	ASF1B
Cluster 1	Clustered	24.66931217	CENPM
Cluster 1	Clustered	24.52272727	AURKA
Cluster 1	Clustered	23.97849462	MYBL2
Cluster 1	Clustered	23.48484848	RAD51AP1
Cluster 1	Clustered	24.52272727	GINS2
Cluster 1	Clustered	24.52272727	CCNB1
Cluster 1	Clustered	21.76086957	PKMYT1
Cluster 1	Clustered	23.53830645	GINS1
Cluster 1	Clustered	23.8172043	CDC25C
Cluster 1	Clustered	23.97849462	TYMS
Cluster 1	Clustered	20.3816092	ORC1
Cluster 1	Clustered	21.18518519	ERCC6L
Cluster 1	Clustered	23.97849462	ZWINT
Cluster 1	Clustered	20.39130435	PCNA
Cluster 1	Clustered	24.74193548	DTL
Cluster 1	Clustered	20.83597884	RFC3
Cluster 1	Clustered	23.92473118	HMMR
Cluster 1	Clustered	23.00689655	UHRF1
Cluster 1	Clustered	22.84666667	SKA1
Cluster 1	Clustered	23.92473118	CEP55
Cluster 1	Clustered	23.68951613	TK1
Cluster 1	Clustered	24.52272727	KIF2C
Cluster 1	Clustered	24.52272727	EXO1
Cluster 1	Clustered	24.52272727	PBK
Cluster 1	Clustered	24.52272727	MCM10
Cluster 1	Clustered	23.03921569	CDC20
Cluster 1	Clustered	23.21746881	RRM2
Cluster 1	Clustered	24.52272727	KIF20A
Cluster 1	Clustered	24.52272727	CDC45
Cluster 1	Clustered	23.77846154	MCM2
Cluster 1	Clustered	22.08730159	POLE2
Cluster 1	Clustered	23.34280303	CDC25A
Cluster 1	Clustered	21.78	CDKN3
Cluster 1	Seed	24.74193548	FOXM1
Cluster 2	Clustered	14.54545455	IL18
Cluster 2	Clustered	12.94586895	CXCL10
Cluster 2	Clustered	13.48529412	TLR1
Cluster 2	Clustered	12.94586895	CCL5
Cluster 2	Clustered	12.94586895	TNF

Cluster 2	Clustered	15.71929825	RNASEH2A
Cluster 2	Clustered	13.76666667	LMNB1
Cluster 2	Clustered	14.38596491	PRIM2
Cluster 2	Clustered	12.86561265	CD68
Cluster 2	Clustered	14.88970588	CDK4
Cluster 2	Clustered	12.87619048	C3AR1
Cluster 2	Clustered	13	IL1RN
Cluster 2	Clustered	12.85380117	TYROBP
Cluster 2	Clustered	12.675	FCER1G
Cluster 2	Clustered	12.94586895	CCR5
Cluster 2	Clustered	13	CXCL16
Cluster 2	Clustered	13.65789474	TNFSF13B
Cluster 2	Clustered	14.70588235	CXCR6
Cluster 2	Clustered	12.94586895	CCL3
Cluster 2	Clustered	12.94586895	FCGR3A
Cluster 2	Clustered	12.94586895	CCL4
Cluster 2	Clustered	12.94586895	ITGAM
Cluster 2	Clustered	14.21052632	VCAM1
Cluster 2	Clustered	14.47368421	CD44
Cluster 2	Clustered	15	IL2RA
Cluster 2	Clustered	14.60784314	CX3CR1
Cluster 2	Clustered	14.88970588	KLRK1
Cluster 2	Clustered	13.85714286	IL7R
Cluster 2	Clustered	13.85714286	CD28
Cluster 2	Clustered	14.88970588	PDCD1
Cluster 2	Seed	15.79084967	TIMELESS

TABLE S5. Detailed primer sets for genes

Primer name		Sequence（5’-3’）	Size（bp）
CCL5	F	CTGCTTTGCCTACATTGCCC	103
	R	TCGGGTGACAAAGACGACTG	
PD1	F	CCCTGGTGGTTGGTGTCGTGG	101
	R	TCCTATTGTCCCTCGTGCGGC	
LAG3	F	AGGCTTCTTGGAGCAGCAGTGTA	202
	R	AGGCAGAAAATCGTCTTGGTCG	
TIM3	F	AGGATGCTTACCACCAGGGGAC	172
	R	CTCCGATGTAGATGCCTATTCTGATG	
CD28	F	CTTTTGGGTGCTGGTGGTGGTTG	171
	R	TAGGGCTGGTAATGCTTGCGGGT	
β-actin	F	TGGCACCCAGCACAATGAA	167

TABLE S6. Detailed information on the web tools

**Web Tool**	**Website**	**Description**
MR Base	http://app.mrbase.org/	MR Base is used to search for GWAS data recorded in the GWAS catalog. The data within this database can directly undergo Mendelian Randomization analysis.
Search Tool for the Retrieval of Interacting Genes Database (STRING)	http://string-db.org/	STRING is a platform used to retrieve known protein interactions and predict interactions between proteins.
GeneMANIA	http://genemania.org/	GeneMANIA collecte hundreds of functional genomics data that can be used for visualizing protein-protein interaction (PPI) networks.
CPTAC cProSite	https://cprosite.ccr.cancer.gov/	CPTAC cProSite is used for cancer proteomic analysis to reveal differences in protein expression between cancerous tissues and normal tissues from different organs.
ADMETlab2.0	https://admetmesh.scbddd.com/	ADMETlab2.0 could assess the absorption, distribution, metabolism, excretion, toxicity and other properties of drugs or small molecules.
AlphaFold Protein Structure Database	https://alphafold.com/	AlphaFold is an AI system developed by Google DeepMind that predicts a protein’s 3D structure from its amino acid sequence.

PART OF FIGURES

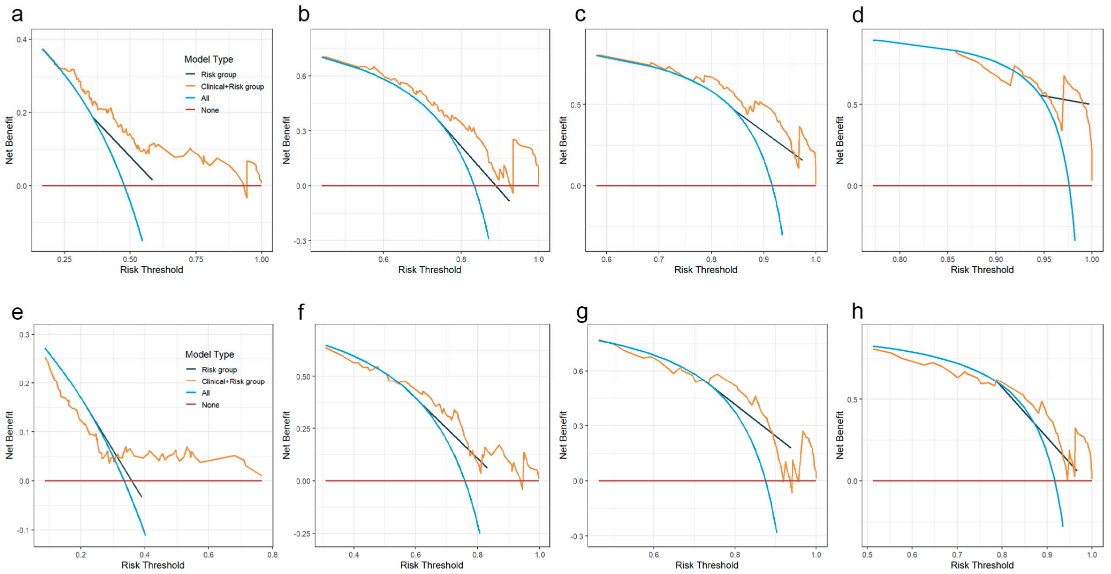

FIGURE S1. Decision curve analysis on four survival models.

(a-d) Decision curves of survival models based on TCGA Set at 400 days, 800 days, 1200 days, and 1600 days, respectively. Dark gray represents survival model includs only risk group, orange represents survival model includs both risk stratification and other clinical information, blue represents the reference that all patients receive intervention, red represents the reference that no patients receive intervention

(e-h) Decision curves of survival models based on CGGA Set at 400 days, 800 days, 1200 days, and 1600 days, respectively. Dark gray represents survival model includs only risk group, orange represents survival model includs both risk stratification and other clinical information, blue represents the reference that all patients receive intervention, red represents the reference that no patients receive intervention

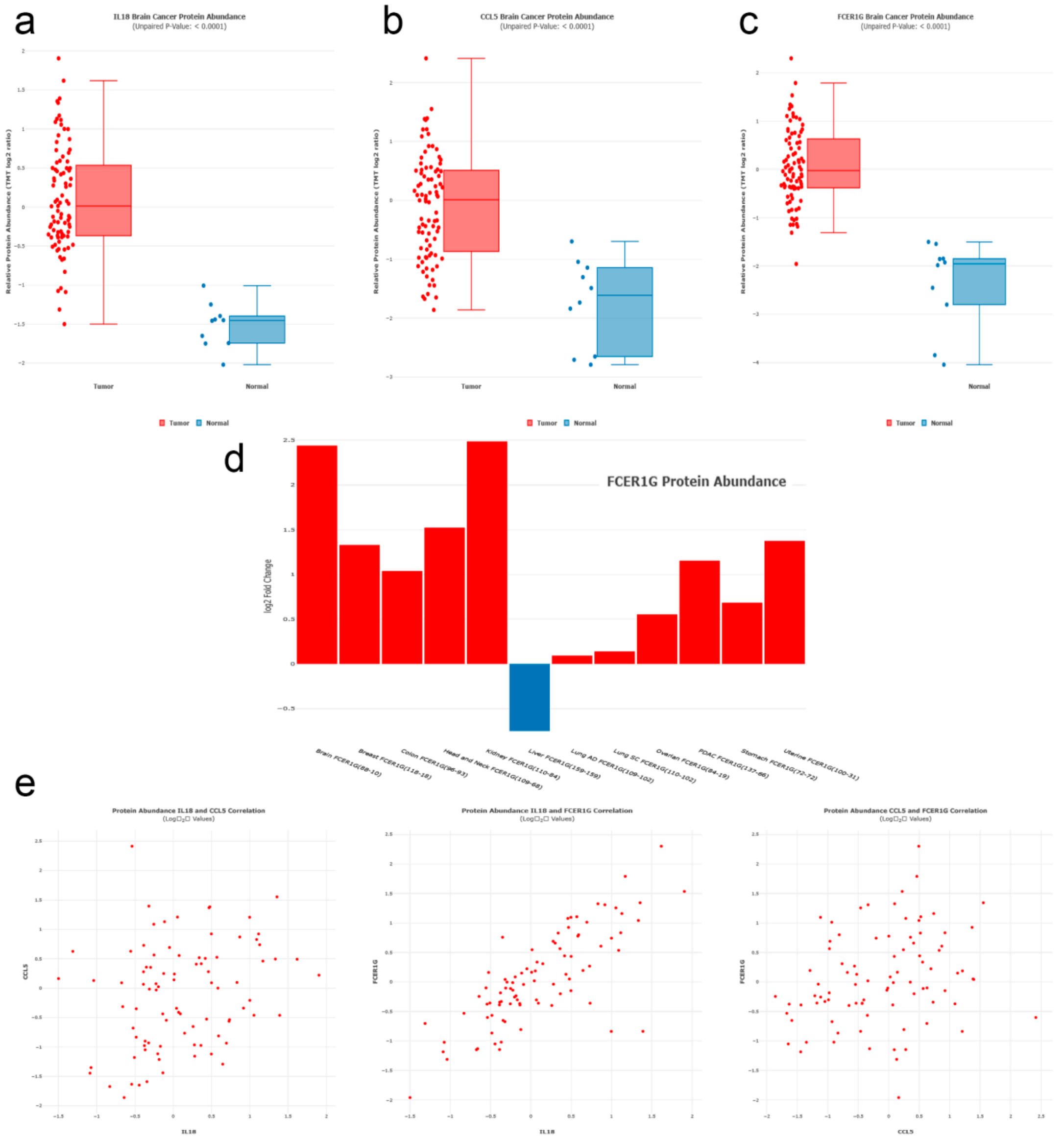

FIGURE S2. Complementary proteomic analysis of key genes.

(a-c) The expression of IL18, CCL5 and FCER1G proteins in brain cancer tissues was significantly higher than that in normal tissues.

(d) Protein expression of FCER1G in pan cancer.

(e) Scatter plot between the expression levels of three proteins (CCL5, IL18, FCER1G).

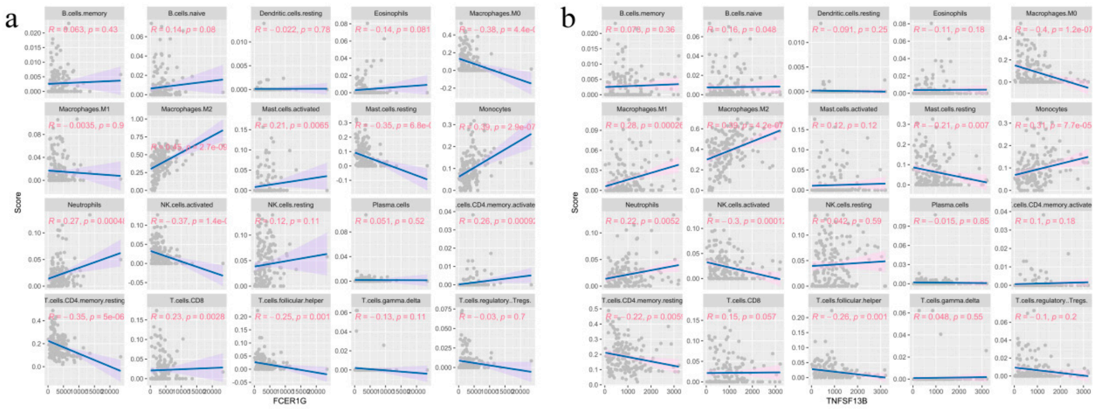

FIGURE S3. Association of various immune cell infiltrations with FCER1G and TNFSF13B

(a-b) The linear relationship between FCER1G, TNFSF13B and tumour-infiltrating immune cells, p-value < 0.05 indicates significant difference.

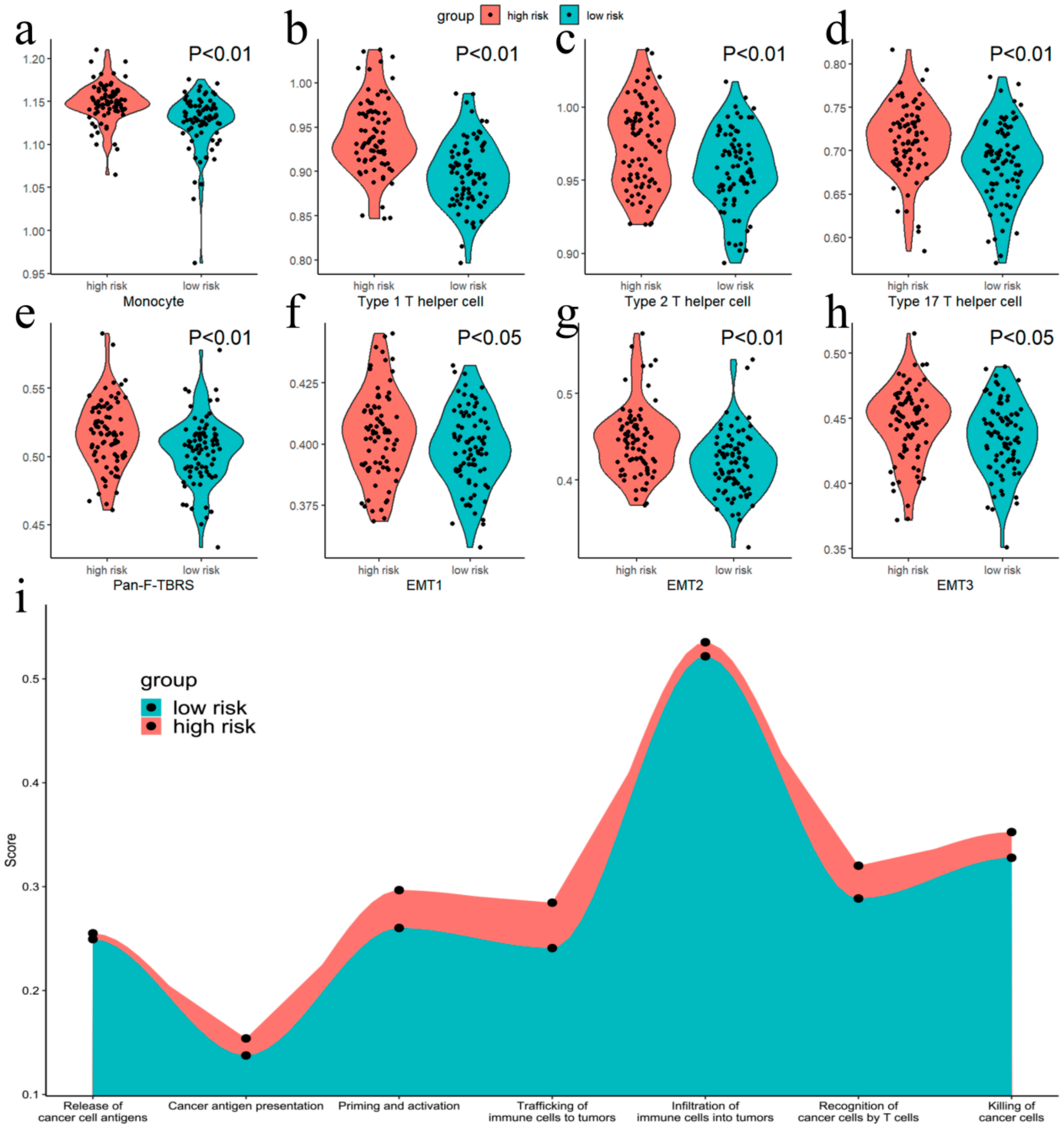

FIGURE S4. The differences between high and low-risk groups in immune cells, immune signaling pathways, and immune cycle.

(a-d) Differences in tumor-infiltrating immune cells (including monocyte and NK cell) between high- and low-risk groups calculated by ssGSEA.

(e-h) Violin plot showing the differences in epithelial-mesenchymal transition related factors between two risk subgroups.

(i) The cumulative line chart of high- and low-risk groups in the Cancer-Immunity Cycle (including release of cancer cell antigens, cancer antigen presentation, priming and activation, trafficking of immune cells to tumors, infiltration of immune cells into tumors, recognition of cancer cells by T cells, killing of cancer cells) of Tracking Tumor Immunophenotype (TIP).

Details of in vitro experiment

1. Chemicals, reagents and primers

TRNzol Universal Total RNA extraction reagent (DP424) and RNase/DNase-free ddH_2O (RT121-02) were purchased from TIANGEN (China). 6×Loading Buffer (D1010) was purchased from Solarbio (China). Trans DNA Marker II (BM411) was purchased from TransGen Biotech (China). Thermo Fisher Scientific (USA) supplied Gibco Dynabead™ Human T-Activator CD3/CD28 magnetic beads (11161D) and RevertAid First Strand cDNA Synthesis Kit (K1622). Applied Biosystems™ MicroAmp™ Fast Optical 96-Well Reaction Plate with Barcode (0.2 mL) and Applied Biosystems™ MicroAmp™ Optical 96-Well Reaction Plate with Barcode & Optical Adhesive Films were obtained from ABI (USA). 2x SYBR Green qPCR Master Mix (B21203) was purchased from Selleck Chemicals (USA).

2. Cell culture and transfection

Well-functioned H9 (derivative of HuT 78) human T lymphocytes (CL0499) were obtained from Pricella (China). The cells were cultured in immune cell culture medium and passaged using dedicated solution for sub-culturing.

The main part of the research was to use CCL5-siRNA (siRNA-323, siRNA-234, siRNA-416) to inhibit the expression of CCL5 in T cells and explore whether inhibiting the expression of CCL5 in T cells can improve the T cell exhaustion. The T cells were divided into three groups: the standard control group, the negative control group (final concentration 10 μM) and the combined CCL5-siRNA (final concentration 10 μM). Gibco Dynabead™ Human T-Activator CD3/CD28 magnetic beads (Thermo Fisher Scientific, USA) was employed for stimulating T cell exhaustion. The exposure duration for each group was 15d. All siRNA used in the transfection were synthesized by Sangon Biotech (China).

3. RNA extraction and real-time qPCR analysis

RNA extraction from cell lysates utilized the RNzol Universal Total RNA Kit, following the manufacturer's instructions. Subsequent to extraction, RNA underwent reverse transcription using the RevertAid First Strand cDNA Synthesis Kit. The resulting cDNA served as a template for amplification with 2x SYBR Green qPCR Master Mix. Gene expression levels were quantified by normalizing to β-actin. Detailed primer sets for genes are provided in Supplementary Table S5.
